# ICTV Virus Taxonomy Profile: *Alphaflexiviridae*

**DOI:** 10.1099/jgv.0.001436

**Published:** 2020-06-11

**Authors:** Jan F. Kreuze, Anna Maria Vaira, Wulf Menzel, Thierry Candresse, Sergey K. Zavriev, John Hammond, Ki Hyun Ryu, ICTV Report Consortium

**Affiliations:** ^1^​ International Potato Center (CIP), Apartado 1558, Lima 12, Peru; ^2^​ Institute for Sustainable Plant Protection – CNR - 73, Strada delle Cacce, 10135 Torino, Italy; ^3^​ Leibniz-Institute DSMZ, Inhoffenstraße 7 B, 38124 Braunschweig, Germany; ^4^​ INRA, Univ. Bordeaux, UMR BFP, CS20032, 33882 Villenave d’Ornon cedex, France; ^5^​ Institute of Bioorganic Chemistry of the Russian Academy of Sciences, 117997 Moscow, Russia; ^6^​ USDA-ARS, USNA, 10300 Baltimore Avenue, Beltsville MD 20705, USA; ^7^​ Seoul Women's University, Seoul, Republic of Korea

**Keywords:** *Alphaflexiviridae*, ICTV, taxonomy

## Abstract

The family *Alphaflexiviridae* includes viruses with flexuous filamentous virions that are 470–800 nm in length and 12–13 nm in diameter. Alphaflexiviruses have a single-stranded, positive-sense RNA genome of 5.5–9 kb. They infect plants and plant-infecting fungi. They share a distinct lineage of alphavirus-like replication proteins that is unusual in lacking any recognized protease domain. With a single exception, cell-to-cell and long-distance movement is facilitated by triple gene block proteins in plant-infecting genera. This is a summary of the International Committee on Taxonomy of Viruses (ICTV) Report on the family *Alphaflexiviridae,* which is available at www.ictv.global/report/alphaflexiviridae.

## Virion

**Fig. 1.**
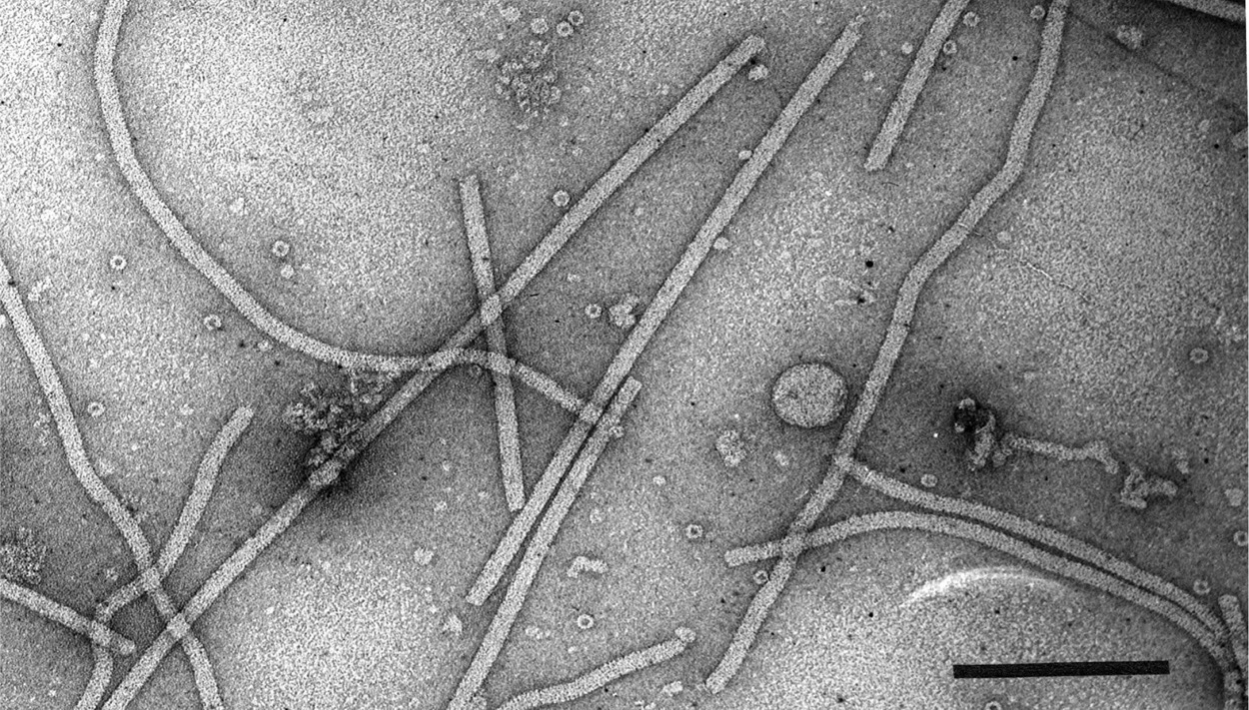
Electron micrograph of negatively-stained virions of an isolate of shallot virus X. Bar=200 nm.

Virions are flexuous filaments, usually 12–13 nm in diameter (range 10–15 nm) and from 470 to about 800 nm in length, depending on the genus. The viral capsid is composed of a single polypeptide ranging in size from 18 to 43 kDa except for members of the genus *Lolavirus,* which have two carboxy-coterminal capsid protein variants, and members of the genus *Sclerodarnavirus* in which no capsid protein has been identified [1, 2] (Fig. 1, Table 1).

**Table 1.** Characteristics of members of the family *Alphaflexiviridae*

Typical member:	shallot virus X, Kanyuka (M97264), species *Shallot virus X*, genus *Allexivirus*
Virion	Flexuous filaments, usually 12–13 nm (range 10–15 nm) in diameter and from 470 to 800 nm in length
Genome	Single molecule of linear, single-stranded, positive-sense RNA of 5.5–9.0 kb
Replication	Cytoplasmic, virus-encoded RNA-directed RNA polymerase
Translation	From capped and polyadenylated genome length and 3′-terminal subgenomic mRNAs
Host range	Plants and fungi
Taxonomy	Realm *Riboviria*, kingdom *Orthornavirae*, phylum *Kitrinoviricota*, class *Alsuviricetes*, order *Tymovirales*; several genera with over 50 species

## Genome

**Fig. 2.**
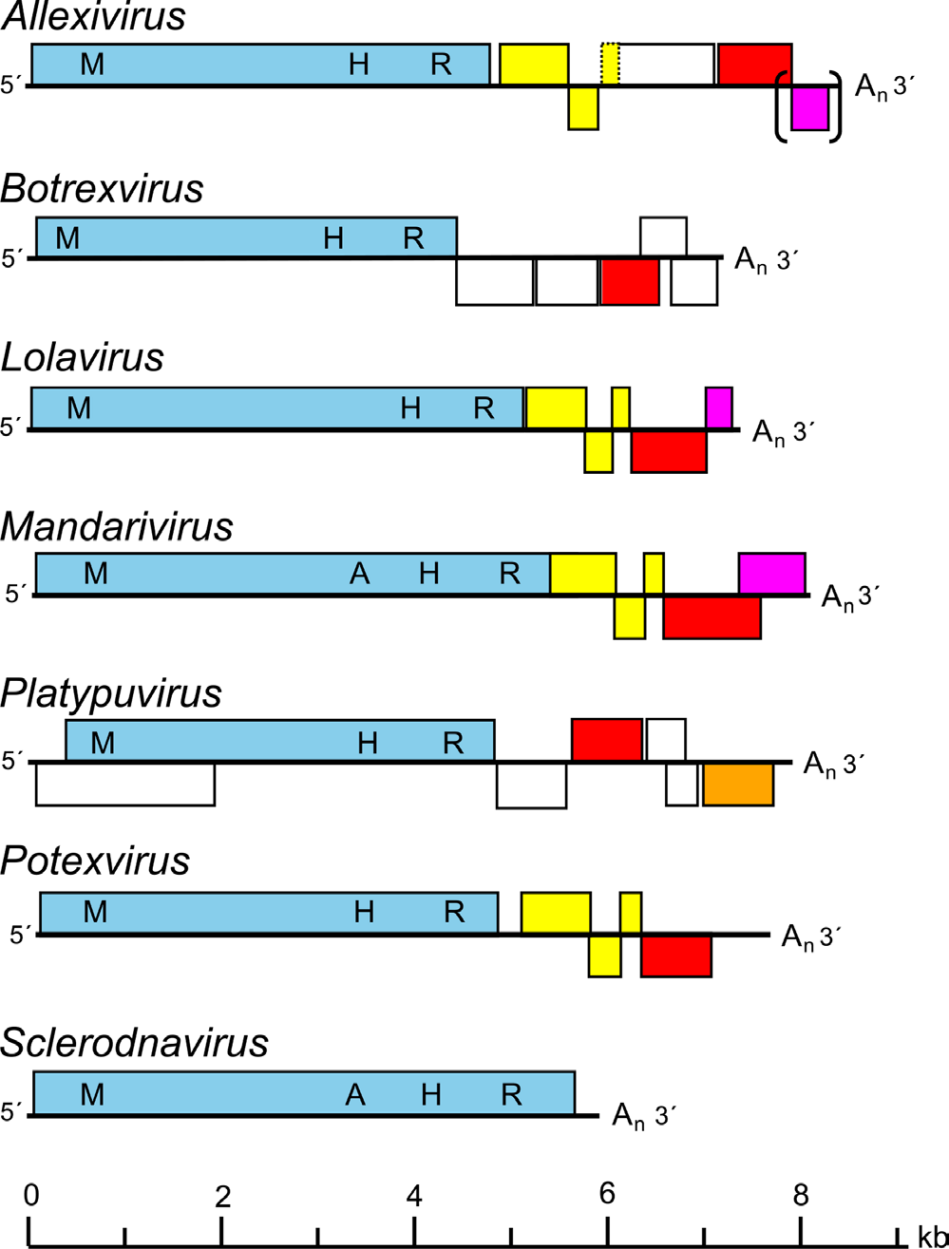
Diagram showing genome organization of members of genera in the family *Alphaflexiviridae*. Blocks represent predicted ORFs. The replicase proteins are shown in blue, triple gene block proteins in yellow, 3A-like movement protein in orange, capsid proteins in red and RNA-binding proteins in purple. Other ORFs are white. The methyltransferase (M), AlkB (A), helicase (H) and RNA-directed RNA polymerase (R) domains of the replicase are also shown. Brackets indicate ORFs may be missing from some members of the genus and a dashed ORF outline indicates a missing start codon.

Virions contain a single molecule of linear, positive-sense RNA of 5.5–9.0 kb, which is 5–6 % by weight of the virion. The RNA is typically capped at the 5′-terminus with m^7^G and has a polyadenylated tract at the 3′-terminus. Smaller 3′-co-terminal sgRNAs are encapsidated in some, but not all, members of the genus *Potexvirus*. There are five to seven genes depending upon the genus, except for members of the genus *Sclerodarnavirus,* which have a single gene. The typical member shallot virus X has six genes (Fig. 2).

## Replication

The protein encoded by ORF1 (or ORF2 in members of the genus *Platypuvirus*) has homologies with replication-associated proteins of the ‘alphavirus-like’ supergroup of RNA viruses [1]. This protein (150–195 kDa) contains conserved methyltransferase, helicase and RNA-directed RNA polymerase motifs [3]; some also include an AlkB domain (alkylated RNA repair protein) [4]. In all plant-infecting members of the family, with the exception of members of the genus *Platypuvirus*, ORFs 2–4 encode the ‘triple gene block’ proteins involved in cell-to-cell movement [5], and ORF5 encodes the viral capsid protein. Some genera have 1–3 additional ORFs downstream of the capsid protein ORF. ORFs downstream of that encoding the replication-associated proteins are translated from 3′-terminal sgRNAs. Replication is cytoplasmic.

## Taxonomy

The family *Alphaflexiviridae* is asssigned to the order *Tymovirales*. The genera *Allexivirus*, *Botrexvirus*, *Lolavirus*, *Mandarivirus*, *Platypuvirus*, *Potexvirus* and *Sclerodarnavirus* include more than 50 species. Alphaflexiviruses infect a wide range of mono- and dicotyledonous plant species. Two members of the family have been discovered that infect the plant pathogenic fungi *Botrytis cinerea* and *Sclerotinia sclerotiorum*, respectively.

## Resources

Current ICTV Report on the family *Alphaflexiviridae*: www.ictv.global/report/alphaflexiviridae
